# The role of adipokines in osteoporosis management: a mini review

**DOI:** 10.3389/fendo.2024.1336543

**Published:** 2024-03-06

**Authors:** Jayaditya Devpal Patil, Salim Fredericks

**Affiliations:** ^1^ Yale New Haven Hospitals- Department of Surgery, New Haven CT, United States; ^2^ The Royal College of Surgeons in Ireland – Medical University of Bahrain, Al Sayh, Bahrain

**Keywords:** adipokines, osteoporosis, chemerin, apelin, leptin, lipocalcin 2, nesfatin-1, visfatin

## Abstract

The prevalence of osteoporosis has been on the rise globally. With ageing populations, research has sought therapeutic solutions in novel areas. One such area is that of the adipokines. Current literature points to an important role for these chemical mediators in relation to bone metabolism. Well-established adipokines have been broadly reported upon. These include adiponectin and leptin. However, other novel adipokines such as visfatin, nesfatin-1, meteorin-like protein (Metrnl), apelin and lipocalin-2 are starting to be addressed pre-clinically and clinically. Adipokines hold pro-inflammatory and anti-inflammatory properties that influence the pathophysiology of various bone diseases. Omentin-1 and vaspin, two novel adipokines, share cardioprotective effects and play essential roles in bone metabolism. Studies have reported bone-protective effects of omentin-1, whilst others report negative associations between omentin-1 and bone mineral density. Lipocalin-2 is linked to poor bone microarchitecture in mice and is even suggested to mediate osteoporosis development from prolonged disuse. Nesfatin-1, an anorexigenic adipokine, has been known to preserve bone density. Animal studies have demonstrated that nesfatin-1 treatment limits bone loss and increases bone strength, suggesting exogenous use as a potential treatment for osteopenic disorders. Pre-clinical studies have shown adipokine apelin to have a role in bone metabolism, mediated by the enhancement of osteoblast genesis and the inhibition of programmed cell death. Although many investigations have reported conflicting findings, sufficient literature supports the notion that adipokines have a significant influence on the metabolism of bone. This review aims at highlighting the role of novel adipokines in osteoporosis while also discussing their potential for treating osteoporosis.

## Introduction

Bone is a tissue with a dynamic structure that is in a constant state of remodeling. This process is governed by osteoclasts and osteoblasts that are modulated by various hormones (1, 2). Osteoporosis (OP) is a common pathology characterized by deficient bone density and deterioration of structural integrity (3). It affects nearly one in three women and one in five men over 50 years globally (4, 5). With a rapidly ageing population and lifestyle modifications, the incidence of OP and OP-related fractures have significantly increased and will continue to rise in the coming years (6). Various factors, both modifiable and non-modifiable, have been linked to OP. Some of these include inadequate nutrition, lack of physical activity, smoking, alcohol, older age, gender, and white ethnic background (7). The predominant clinical consequence of OP is fragility fractures, where OP is responsible for more than 9 million fractures annually across the globe, with the highest incidence in Europe (34.8%) (8).

OP and its associated complications impact the health of the patient affected as well as their quality of life. Further, OP impacts healthcare systems (9). The financial burden associated with OP in the EU (2010) was estimated to be 37 billion Euros. The bulk of which, an excess of 70%, was linked to bone fractures. In the US (2005), the direct medical costs of OP were estimated to be 14 to 20 billion US dollars. Projecting forward to 2025, there is likely to be over three million cases of osteoporotic fractures annually. This comes with an estimated price tag of 25 billion US dollars (10, 11).

Pharmacological treatments for OP historically centred upon decreasing osteoclastic bone resorption or increasing osteoblast activity. The biochemical and cellular mechanisms of action of these drugs employ varied and complex pathways. Examples include enhancing apoptosis, inhibiting cholesterol synthesis within osteoclasts, and perturbing the nuclear factor kappa-B (NFκB) system to inhibit osteoclast function along with their formation. Many of these mechanisms of action are still being elucidated. Adipokines assume both endocrine and paracrine roles that regulate an array of processes. These include energy regulation, metabolism, inflammation and cardiovascular function (12). These chemical mediators have been shown to influence the known modes of action of current anti-OP drugs for treating osteoporosis. Moreover, adipokines have also been shown to directly influence bone metabolism as well as bone density (12). These findings pose the question: will a novel treatment for osteoporosis emerge based on our knowledge of adipokines? Pre-clinical studies suggest that adipokines have essential roles in regulating bone homeostasis. Clinical studies have shown that more than 95% of adult OP patients have at least one comorbidity, and more than 60% have three or more comorbid diseases (13). These include diabetes, metabolic syndrome and obesity. These all have well-established associations with serum adipokine concentrations. Clinical studies have shown a significant relationship between adipokine plasma concentrations and bone strength parameters such as bone mineral density (BMD), cortical thickness, and fractures. Thus, there may be significant prognostic importance to measuring adipokines. Hence over the last 15 years several reports have appeared relating circulating levels of adipokines to measures of bone strength in various patient groups. The first of these circulating factors to be discovered (leptin and adiponectin) had been shown to play a role in bone metabolism and BMD modulation (14). These newly discovered adipokines have been shown to have both positive and negative influences on BMD. This mini review aims to highlight the role of novel adipokines in influencing bone metabolism and their potential for treatment of OP (**Table 1**).

**Table 1:** Adipokines and bone metabolism and the potential relevance to osteoporosis.

Adipokine	Role in bone metabolism	
	*In vitro*	*In vivo*
Apelin	Apelin-13 is involved in inflammatory processes and programmed cell death of bone marrow stromal cells (15).	Lower levels of apelin-13 are associated with osteoporosis (16, 17).
Chemerin	Inhibits osteoblast activity (18, 19).	Plasma chemerin concentrations are elevated with osteoporosis (20).
Leptin	Promotion of osteogenesis (21, 22). Stimulation of the differentiation of stromal cells into osteo-blasts (23), induces an increase in the proliferation of osteo-blasts and inhibits production of osteo-clasts (24).	Increases bone mass by increasing bone formation and inhibiting bone resorption (25).
Lipocalin-2	Overexpression of *LCN2* gene stimulates the production of RANKL and IL-6, resulting in increased osteoclastogenesis and reduced osteoblast differentiation (26).	Correlation between plasma concentration and risk of fracture (27).
Metrnl	Increases osteoblast activity (28).	Plasma concretions are associated with established osteogenic molecules (29).
Nesfatin-1	Induces pro-inflammatory agents primary chondrocytes (30).	Protective of bone tissue in animal studies (31, 32).
Omentin-1	Inhibits osteoclast activity (33).	High plasma omentin-1 is linked with the development of OP in female diabetics (34).
Resistin	Plys a role in the pathogenesis of bone defects (35).	High plasma resistin is associated with increase in osteoclastic activity (36). Negatively affects bone metabolism by increasing osteoclastogenic activity (37).
Visfatin	Proliferation of osteoblasts (38).	No clear understanding of the influence of visfatin on bone strength (39–41).

### Apelin

Adipocytes produce the peptide apelin and secret it into the blood (42). Several active forms of this adipokine exist, with apelin-13 and apelin-36 being the most important physiologically (43). Pre-clinical studies have suggested that apelin supports bone metabolism regulation. Apelin-13 may contribute to the protection of bone by influencing inflammatory processes and programmed cell death of bone marrow stromal cells, which are implicated in bone strength (15). Thus, apelin promotes the genesis of osteoblasts and inhibits their destruction. Clinically, apelin was found not to be a significant independent predictor of the strength of bone (44). Lower levels of apelin-13 have been reported in patients with osteoporosis compared to those found in non-osteoporotic individuals (16, 17). Little to no literature was available that exclusively studied the role of apelin in directly treating or preventing OP.

### Chemerin

Chemerin was first identified as a gene product of *tazarotene-induced gene 2 (TIG2)*, whose expression was up-regulated in psoriatic skin in response to synthetic retinoids (45). Historically, it has been referred to by the names tazarotene-induced gene 2 (TIG2) and retinoic acid receptor responder 2 (Rarres2) (45). The protein product of this gene is highly expressed in adipose tissue, lung and liver (46, 47). It plays a role in metabolic and inflammatory pathologies has been widely discussed (46, 48). Recent studies have highlighted the role of chemerin in OP. The null expression of chemerin protein or it’s receptor (chemokine-like receptor 1; CMKLR1) in a knockout mouse model has been associated with pro-bone strengthening processes. These include the upregulation of osteoblast marker gene expression and mineralization of bone following exposure to osteoblastogenic stimuli. Chemerin/CMKLR1 has also been shown to alter the expression of certain transcription factors associated with osteoclastogenesis, thus limiting osteoblastogenic Wnt signaling (49). Pre-clinical data has shown increased bone loss in db/db mice that induced increases in levels of chemerin and CMKLR1 expression. Additionally, treatment with CMKLR1 antagonists inhibit bone loss (50). This suggests a role for chemerin antagonists as potential therapeutic agents for osteoporosis. Han L et al., demonstrated that a lack of chemerin is associated with increased expression of genes linked to osteoblastogenesis and inhibition of genes linked to osteoclastogenesis pathways in the cell lines MC3T3-E1 and Raw264-7. The authors suggest that achieving low circulating concentrations of chemerin may be an appropriate therapeutic strategy in the treatment of OP (18).

A case-control study assessing the relationship between chemerin levels and OP and BMD, described an inverse correlation between serum chemerin concentration and bone density, as assessed by BMD at the femoral neck and lumbar spine (51). Similar findings were reported in another study involving obese post-menopausal women, which showed serum chemerin concentrations to be inversely related to BMD of lumbar vertebrae. This study from China carefully controlled for age as well as lean and fat mass (52). A report of a population study from Northern Europe showed that serum chemerin concentrations were negatively correlated with bone strength exclusively in a group of obese individuals of both sexes and not for those who were overweight and lean. This study from Pomerania also demonstrated an association between OP risk and serum chemerin concentrations (53).

Similarly, another population study examining serum concentrations of chemerin in women with post-menopausal OP reported chemerin to be significantly lower in the women with OP than controls with no OP. These findings suggest that chemerin, along with other adipokines, may play important roles in the pathogenesis of OP, although no direct correlation was studied (54). Menzel J et al. investigated the association between chemerin and broadband ultrasound attenuation (BUA), as a surrogate indicator of bone health in pre-menopausal, peri-menopausal, and post-menopausal women. The results showed an inverse relationship between chemerin and BUA in the pre-menopausal and peri-menopausal groups. The study suggested high chemerin levels may minimize peak bone mass, subsequently promoting age-related bone loss. However further exploration of this area was recommended (55).

Chemerin has also been linked to altered BMD in a patient group diagnosed with inflammatory bowel disease (IBD). One study reported serum concentrations of chemerin, visfatin, and vaspin in relation to BMD in patients with IBD. Results showed elevated levels of chemerin in patients with IBD and an independent correlation was observed with chemerin serum concentrations. The authors suggested a role for both chemerin and visfatin in the pathogenesis of OP associated with IBD. However, further analysis of pathways was not performed (56). A study that aimed to elucidate upon the potential position of plasma chemerin in the prognostication of OP fractures showed a negative correlation between circulating chemerin concentrations and BMD. The sites assessed in that study were the femoral neck and lumbar spine. The study also reported higher plasma concentrations of chemerin in patients with osteoporotic fractures relative to healthy controls. This suggests that chemerin may be a valuable circulating soluble surrogate marker for predicting OP-associated fractures. A more refined analysis of the data, considering body mass index and age, revealed a considerably stronger predictive value for plasma chemerin. The study described an important role for chemerin in regulating the metabolism of bone. The authors posited processes underpinning fractures associated with OP involving chemerin. Comprehensive investigations of chemerin in this setting are essential for chemerin to claim its diagnostic and prognostic status as a clinically relevant circulating segregate marker with OP (20).

A study analyzing the relationship between chemerin and bone metabolism, whilst accounting for the RANKL/RANK/OPG system, in girls with anorexia nervosa showed that chemerin acts to balance the dynamic interactions between bone metabolism and the OPG/RANK/RANKL system. Chemerin may influence the reduction in BMD associated with anorexia nervosa. The study also found chemerin to exert more severe action at cortical bone sites compared to trabecular bone sites (57). Serum chemerin levels in response to OP treatments have also been addressed. One study assessing the changes in plasma concentrations of chemerin, vaspin, omentin-1, and osteoprotegerin flowing treatment with ibandronate in post-menopausal patients with OP found a decrease of 24% following six months of treatment. Thus, suggesting an increase in circulating chemerin may be implicated in OP (58).

Numerous studies have supported an inverse relationship between chemerin plasma concentration and BMD. These findings have been replicated in both pre-clinical and clinical studies. Although some literature has assessed chemerin serum levels in response to OP therapy, no study has directly evaluated the effects of chemerin suppression in OP or in patients at risk of OP.

### Leptin

Leptin was the first of the adipokines to be discovered and investigated. Predominantly, leptin is produced by adipocytes (59). Leptin receptors serve as an indicator of a bone marrow-derived mesenchymal stem cell (BM-MSC) subpopulation and has been shown to promote osteogenesis via JAK/STAT signaling pathways (21, 22). It also plays a role disease-inducing and disease-progression of: cardiovascular disorders, type-II diabetes mellitus, and malignancies (60). Although studies support leptin to have a positive impact on BMD, negative effects have also been reported. *In vitro*, leptin has been shown to stimulate the generation osteoblasts from stromal precursor cells (23). Leptin induces the cell growth of osteoblasts and attenuates osteo-clastogenesis, whilst having no influence on osteoclasts in their mature state (24). *In vivo* studies have suggested that pharmacological administration of leptin, peripherally may lead to a strengthening of bone by increasing the formation of bone and reducing the resorption of bone (25). In contrast, intracerebroventricular administration of leptin results in bone loss in leptin-deficient and wild-type mice (61). The sympathetic branch of the autonomic nervous system is thought to mediate this bone-weakening effect of central admiration of leptin (62).

Human studies have reported conflicting results regarding leptin and OP. One study analyzing the relationship between either circulating levels of leptin or the percentage of body fat versus measures of BMD. Leptin concentrations were further correlated with the presence or absence of vertebral compression fractures. Circulating leptin was found to correlate with BMD across several skeleton sites. This positive correlation was maintained even after applying a correction for body fat and age (63). In a US population study, Ruhl et al. analyzed levels of serum leptin in relation to BMD in pre-menopausal women and post-menopausal women and found no association of between the two (64). Such conflicting findings challenge the role of leptin in BMD which in turn determines OP. The osteogenic potential of BM-MSC is thought to be influenced by leptin production. This intriguing notion is evidenced by the finding that BM-MSCs that overexpress leptin have a higher capacity for osteogenic differentiation relative to wild-type cells (65, 66). Projecting forward the genetic modification of BM-MSC to overproduce leptin offers the potential for improving therapeutic approaches for patients with OP (67).

### Lipocalin-2

Lipocalin-2 is encoded by the *LCN2* gene in humans. Structurally, it is a glycoprotein, and functionally, it is a plasma protein responsible for transporting small and hydrophobic molecules to target following interaction with megalin/glycoprotein and GP330 SLC22AL7 or 24p3R LCN-2 receptor (68). Lipocalin-2 has been implicated in many physiological and pathological functions (69).

A large prospective cohort study investigating plasma lipocalin-2 concentrations in relation to fracture-related hospitalization in elderly women (above 70 years) reported a strongly positive correlation between circulating levels and risk of fracture and OP (27). This study suggested a significant relationship between subjects’ lipocalin-2 levels and osteoporotic fractures. Another study investigated the relationship between plasma lipocalin-2, markers of bone turnover and BMD in 1012 women (age 20-88) assessing the lumbar spine and femoral neck. They reported lipocalin-2 concentrations to be inversely correlated with BMD (70). This study found no significant relationship with osteoporotic fractures. A major difference between the two studies was that one involved Western Australian women (3), and the other involved mainly Chinese women (70). East Asians are known to have low fracture rates despite having lower BMD (71). Thus, a more inclusive and representative study should be conducted to determine a more informed conclusion and analyze the differences between different age groups and races.

Lipocalin–2 may be involved in the development of OP. One study investigating the role of lipoclin-2 as a potential therapeutic target in Duchenne muscular dystrophy (DMD) -induced bone loss in mice reported that deleting *Lcn2*, (by downregulation or blocking its activity) attenuated the loss of bone associated with DMD. This suggests lipocalin inhibition plays a role in offsetting OP onset (72).

Murine and human studies have shown that lipoclin-2 expression is responsive to a lack of mechanical stimulation from bone and skeletal muscle. It is up-regulated when exposed to mechanical underload (26, 73, 74). Surprisingly, a lack of lipoclin-2 is detrimental to the health of bone, as it acts indirectly upon osteoblasts through modulating metabolism (75). Overexpression of the *LCN2* gene stimulates RANKL and IL-6 production. In turn, this induces osteoclastogenesis and reduces osteoblast maturation (26).

### Meteorin-like protein (Metrnl)

Meteorin-like protein (Metrnl) was initially identified as having properties similar to those of the neurotrophic factor - Meteorin. It has physiological roles in inflammation and glucose metabolism and is abundantly expressed by muscle tissue (76). The close anatomical proximity of muscle and bone has led to the discovery of interesting common pathological mechanisms between the two tissues (77). This mechanical and chemical cross-communication within the tissues of the musculoskeletal system may have particular significance during ageing and the development of age-related illnesses. Metrnl is thought to be a key common molecule affecting both BMD and muscle atrophy (78). The functions of Metrnl are yet to be understood entirely. However, it has been reported to be associated with lipid homeostasis (79), chronic obstructive pulmonary disease (80), coronary artery disease (81), and insulin resistance (82).

Huang et al. described trends in the skeletal expression of Metrnl during pre-natal and post-natal development and the healing of fractures. They reported Metrnl ‐deficiency to have no detectable effect on fracture healing. The study also showed that doubling Metrnl expression within the fracture callus did not significantly affect bone healing (28). Overexpression of Metrnl has also been suggested to inhibit the mineralization of nodule formation (83). Cherian et al. (29) observed a strong positive correlation between circulating Metrnl concentrations and well-established molecules with osteo-genic properties in obese and diabetic patients. This suggests that Metrnl affects complications associated with bone development.

Although Metrnl may influence osteoblasts *in vitro* and *in vivo*, considering the broad effects of Metrnl on inflammation and muscular physiology, studies suggest that Metrnl mediates its effects via macrophages. The addition of Metrnl at cellular levels potentially improves osteoblast activity (84).

Other studies failed to find significant findings suggestive of a specific role for Metrnl in bone metabolism. One study reported Metrnl‐deficiency to have no detectable effect on fracture healing, and conversely, doubling the expression of Metrnl within the fracture callus had no change in bone healing (28). The osteo-inductive nature of Metrnl may prove useful for better designing treatment strategies for OP. Studies have proposed Metrnl supplementation to be potentially effective in fracture healing via osteo-inductive properties however, further trials will be required to confirm such an association, map pharmaco-kinetics and summarize therapeutic doses.

### Nesfatin-1

Nesfatin-1 is a pleiotropic bioactive peptide associated with satiation and appetite regulation (85). Nesfatin-1 is expressed ubiquitously, and a major serum source is white adipose tissue. Early on it was identified as anorexigenic and this neuroendocrine function has been a key research focus (86). Nesfatin-1 has been shown to influence the production of pro-inflammatory mediators, e.g. COX-2, IL-6 and IL-8 in human primary chondrocytes in patients with osteoarthritis (30); it may play a role in pathogenesis or osteoarthritis, although the exact pathogenesis remains undefined (30, 87, 88). Pre-clinical studies have shown nesfatin-1 to be protective of bone tissue in rodents (31, 32). Thus it was suggested that this adipokine may be used therapeutically in the management of OP (89). Nesfatin-1 stimulates the release of pro-inflammatory mediators from chondrocytes cultured from patients with osteoarthritis. Serum and synovial fluid concentrations have been shown to have a direct relationship with the disease-severity of osteoarthritis (30, 87, 88). It is clear that more basic and clinical research is needed to develop an understanding of nesfatin-1’s role in bone degradation.

### Omentin-1

Omentin-1, or intelectin-1 (ITLN1), is an adipocytokine extensively expressed in human visceral fatty tissue. Plasma concentrations have been shown to be inversely related to the levels of pro-inflammatory factors in patients with impaired glucose regulation and chronic artery disease (90, 91). Omentin-1 has been shown to attenuate the formation of atherosclerotic lesions by reducing the macrophage-mediated inflammation (92). Despite association with other pathologies, mixed results have been reported on the role of omentin-1 in OP pathogenesis. Increases in pro-inflammatory cytokines associated with chronic inflammation contribute to the loss of bone by increasing osteoclast differentiation and inhibiting osteoblast activity (93). Osteoblasts are direct omentin targets via the PI3K/Akt signaling pathway (94). A murine demonstrated that omentin-1 inhibited osteoclast differentiation *in vitro* via an indirect mechanism involving the stimulation of the production of the soluble glycoprotein osteoprotegerin (OPG) and reducing the secretion of receptor activator for NFκB ligand (RANKL) in osteoblasts (33). Further, the study reported upon *in vivo* findings that intravenous administration of omentin-1 (delivered via a viral vector) prevents loss of bone and bolsters bone strength in an ovariectomy-induced OP mouse model and in an OPG-deficient mouse model (33, 95). Thus, omentin-1 potentially inhibits the effects of inflammation-induced OP by simultaneously addressing metabolism within bone and inflammation.

Similar findings were reported upon in a study assessing omentin-1 depletion and its effects on pro-inflammatory cytokines and bone destruction. Rao et al. demonstrated that omentin-1 has a central role in maintaining of healthy bone mass, alleviating inflammation and reducing loss of bone in OP. The mechanism that underpins this is probably the downregulation of pro-inflammatory mediators. Omentin-1 has the ability to inhibit inflammatory responses emanating from macrophages activation and reduce their anti-osteoblastic and pro-osteoclastic activities. Thus it is suggested that omentin-1 has a potential use in preventing and treating inflammatory bone diseases (96).

The associations between plasma omentin-1 concentration, BMD, fragility fractures, and various other relevant measures has been investigated in a Chinese population. It concluded that high omentin-1 plasma concentration is potentially associated with OP development in female diabetic patients. Thus, omentin-1 is a candidate surrogate serum marker for diabetic OP among females. Further, multivariate analysis showed plasma omentin-1 to be an independent, decisive factor for OP in women exclusively (34).

Another study assessing circulating levels of omentin and vaspin and their role in BMD in multiple sclerosis (MS) patients and healthy controls reported raised omentin-1 plasma concentrations correlated with BMD, and circulating concentrations of osteocalcin and osteopontin in patients with MS. This relationship between omentin-1 and bone in MS, which is an autoimmune disease, indicates crosstalk between adipose, bone, and cells of the immune system. They all share several communication molecules acting as mediators and messengers in a complex web of interactions. The charting and elucidation of the intertwining of bone and adipocytokines complex systems may provide new pharmacological approaches for tackling the treatment of osteoporosis as well as autoimmune states such as MS (97).

Some studies contradict the protective effects of omentin-1. Tohidi et al., assessed plasma concentrations of omentin-1 in relation to BMD in post-menopausal women. They reported that plasma omentin-1 had a negative correlation with BMD. This was regarding lumbar spine site post-menopausal women in an Iranian population sample (98). Another study investigated the relationship between plasma omentin-1 and vitamin D with BMD in post-menopausal women with OP. Compared with an age-matched non-osteoporotic control group, plasma omentin-1 concentrations tended to be higher in post-menopausal women than controls, but this increase in plasma concentration was only significant in women with OP. There were no significant differences in plasma vitamin D concentrations between the groups. When women were sub-categorized based on plasma vitamin D concentrations, those with normal plasma vitamin D had higher omentin-1 concentrations. A weak but statistically significant positive correlation was shown between omentin-1 and vitamin D concentrations across all groups (99).

Another study investigated the relationship between circulating omentin-1 concentrations and the presence of OP in older men and found that omentin-1 was an independent predictor of BMD in elderly men with OP, and it was negatively correlated with markers of bone turnover. This suggests that omentin-1 has a negative effect on bone mass in older men with OP (100). Another study, examining plasma concentrations of chemerin, vaspin, omentin-1, and osteoprotegerin following treatment with ibandronate in post-menopausal patients with OP found omentin-1 concentrations to have a positive relationship with BMD in post-menopausal individuals. Omentin-1 also predicted bone strength in the hip bone (58).

### Resistin

Resistin is a 12.5 kDa peptide hormone, rich in cysteine that is expressed in adipocytes, monocytes and bone marrow cells (101). It is known to possess pro-inflammatory properties and has a negative influence on the metabolism of bone metabolism, achieved by stimulating osteoclastogenic activity. Multivariate analysis has shown it to be the independent predictor of poor bone strength (37). High circulating resistin concentrations are associated with high osteoclastic activity, which is linked to its role as a pro-inflammatory mediator (36). It has a role in the remodeling of bone by inducing osteoclastogenesis. It promotes resorption via the NF-κB pathway activation (102).

One study investigating the association between plasma vitamin D resistin concentrations in post-menopausal, non-osteoporotic and osteoporotic women found increasing concentrations of resistin in plasma in post-menopausal osteoporotic individuals (103). Other studies have also reported elevated plasma resistin concentrations in menopausal females with OP when compared to a control-group. This could potentially be due to increased pro-inflammatory cytokines such as IL-1 and IL-6. This may be the primary contributor in the OP pathogenesis. It has also been showed that bone metabolism in post-menopausal women is significantly governed by levels of resistin, leptin, IL-1, IL-4, IL-6, and TGF-β. These could be used diagnostically and prognostically as biomarkers in identifying patients at risk of OP and its associated bone fracture-risk (36).

A further means by which resistin influences bone metabolism is via its effect on the bone-derived protein hormone osteocalcin. It is considered a viable candidate marker of bone formation as it is secreted by osteoblasts. Circulating resistin concentration is negatively correlated with osteocalcin. Low osteocalcin levels are associated with high resistin levels in OP (104). This suggests that resistin regulates osteocalcin by a mechanism of negative feedback, i.e. resistin suppresses osteocalcin production and is released in response to low osteocalcin levels (104).

One study exploring the potential role of resistin in osteogenic differentiation and pathways involved showed the processes of osteogenic differentiation induced by resistin may be related to triggering the PI3K/AKT/mTOR pathway. This triggering response is reduced by inhibitors of PI3K/AKT/mTOR. This suggests that resistin is implicated in the development of bone disease. Which, in turn, highlights the therapeutic potential of the resistin system in OP (35).

### Visfatin

The pro-inflammatory adipokine visfatin is expressed in several tissue types (105, 106), with adipose tissue being the main source (107). It is a 52 kDa enzyme formally known as Nampt (nicotinamide phosphoribosyltransferase) because of its role in the conversion of nicotinamide to nicotinamide mononucleotide (NMN) in the NAD salvage pathway (108). Pre-clinical experiments have highlighted the position of visfatin in the process of the proliferation of osteoblasts. In common with other adipokines, visfatin is involved in inflammatory responses, bone- catabolism, and more specific influences in osteoblastic glucose uptake and collagen synthesis. An association has been reported between visfatin and inflammation-mediated via toll-like receptor 4 (TLR4) (38). There is a disparity in the clinical literature regarding a correlation between visfatin plasma concentrations and measures of bone strength (39–41). However, associations have been reported between visfatin and obesity type II diabetes (109, 110). Both obesity and diabetes are established risk factors for OP. It has been suggested that serum visfatin may be a surrogate biomarker of obesity (111), as visfatin circulating concentrations are increased in obesity (112). Elevated plasma visfatin concentrations are linked to inflammatory states, which may lead to pathological metabolic syndromes. Conditions such as diabetes mellitus and insulin resistance have several associated comorbidities, which include OP. As the intricate relationship between inflammation, deranged metabolic states and OP is elucidated, visfatin will likely emerge as a key contributor to these pathogenic intricacies. However, before clinical benefit can be obtained from the mapping out of visfatin’s pathophysiological functions, more research is required in this area. The tools are now available for this research to progress apace. Several visfatin inhibitors are now available; three of these inhibitors, CHS828, FK866 and KPT-9274 have been investigated in clinical trials (113) and are likely to spearhead clinical pharmacological research in the coming years.

## Conclusion

Understanding the position of novel adipokines in the prognosis of OP is vital for allowing researchers and clinicians to study treatment and preventative modalities. Current literature has highlighted associations between certain adipokines and bone loss with various modalities. Within the musculoskeletal system, adipokine research has started to reveal a dynamic relationship between muscle and bone at the biochemical level. Furthermore, chemical communications between diverse tissues, such as adipose tissue and bone, are starting to be elucidated. Adipokines also provide a common pathophysiological thread between illnesses of the elderly. However, to assess the therapeutic potential for adipokine in OP management, we suggest more targeted pre-clinical trials with extension into human trials. Indeed, research has shown that OP cannot be understood isolated from other comorbidities such as proinflammation states, diabetes and obesity, all of which affect BMD. These conditions are intertwined, from their pathogenesis to their clinical presentation. Unsurprisingly, adipokines are implicated in all aspects of these states. These chemical mediators are involved in the three-way dynamic interactions between adipose tissue, bone tissue and immune cells. They may prove pivotal in developing new effective treatments not only for OP but also for diabetes and chronic inflammation.

## Author contributions

JP: Conceptualization, Data curation, Formal Analysis, Investigation, Methodology, Resources, Writing – original draft, Writing – review & editing. SF: Conceptualization, Data curation, Funding acquisition, Investigation, Methodology, Project administration, Supervision, Writing – review & editing.
